# Impact of nosocomial infections on neurodevelopmental outcome and rehospitalization rate in preterm infants with birth weight below 1500 g (NINO study)

**DOI:** 10.1038/s41372-026-02681-2

**Published:** 2026-05-05

**Authors:** Elisabeth Resch-Poteralski, Ute Maurer-Fellbaum, Julia Eichberger, Volker Strenger, Robert Krause, Elisabeth Pichler-Stachl, Alexander Avian, Bernhard Resch, Berndt Urlesberger

**Affiliations:** 1 Division of General Pediatrics, University Clinic for Pediatrics and Adolescent Medicine, Medical University of Graz, Graz, Austria; 2 Division of Neonatology, University Clinic for Pediatrics and Adolescent Medicine, Medical University of Graz, Graz, Austria; 3 Outpatient Clinic of Neurodevelopmental Follow-up, University Clinic for Pediatrics and Adolescent Medicine, Medical University of Graz, Graz, Austria; 4 Department of Infectious Diseases, University Clinic for Internal Medicine, Medical University of Graz, Graz, Austria; 5 Institute for Medical Informatics, Statistics and Documentation, Medical University of Graz, Graz, Austria

**Keywords:** Outcomes research, Bacterial infection, Viral infection

## Abstract

**Objective:**

This study aimed to determine the influence of nosocomial bacterial and viral infections of preterm very low birth weight (VLBW) infants on neurodevelopmental outcome and on rehospitalization rates during the first two years of life.

**Study design:**

Retrospective single-center cohort study including preterm infant born between 2010 and 2018 and followed until two years of age corrected for prematurity (Bayley scales).

**Results:**

Of 620 study infants 418 without neurodevelopmental impairment (NDI). were compared to 202 infants with NDI. Single or multiple nosocomial infections were not risk factors for NDI in a multivariate logistic regression model checked for multicollinearity. Infants with NDI were of younger gestational age, had lower birth weights and higher rates of neonatal complications (ileus, periventricular leukomalacia, and bronchopulmonary dysplasia). Nosocomial infection were also not risk factors for rehospitalizations by infectious diseases.

**Conclusions:**

Nosocomial infections did not significantly influence NDI and rehospitalization rates in preterm VLBW infants.

**Trial registration number:**

The study was registered at “Deutsches Register Klinischer Studien” DRKS00019000.

## Introduction

Neonatal sepsis is associated with substantial morbidity and high rates of mortality especially in the very low birth weight infant (VLBW, <1500 g). The responsible pathogen might be associated with chorioamnionitis, might result from maternal flora, or might arise from a postnatal infection acquired from the hospital or community. The timing of exposure to the pathogen, the bacterial load, the neonatal host reaction by its immune system, and virulence of the pathogen all contribute to the clinical presentation of neonatal sepsis [1]. Immunological immaturity, as it is the case in preterm infants, aggravates the course of the disease resulting in prolonged hospital stays. Moreover, the need for invasive procedures in this high-risk group of vulnerable infants places them at high risk for nosocomial infection [1]. The limited number of studies that describe the long-term consequences of neonatal infections, often undertaken in high-income settings and using variable designs and diagnostic tools, were not sufficient to inform clinical practice and policy prioritization [2]. However, the risk of NDI after neonatal bacterial meningitis is higher than the corresponding risk after neonatal sepsis [2].

Neurodevelopmental outcome of very low birth weight infants is determined by overall immaturity related to an extremely low gestational age, low birth weight, socioeconomical factors, and complications associated with interventions administered in the NICU. In a recent Canadian study gestational age, sex, outborn, illness severity, bronchopulmonary dysplasia (BPD), necrotizing enterocolitis (NEC), late-onset sepsis, retinopathia of prematurity (ROP), abnormal neuroimaging and site of the neonatal unit were statistically significant associated with moderate to severe neurodevelopmental impairment (NDI) [3].

To what extent neonatal sepsis contributes to the infant´s outcome is less well known. Inflammation has become a critical factor for normal development or injury in the immature brain. Preterm birth, as mentioned, is often a result of exposure to inflammation at a very early stage of development. Hence, it possibly affects the brain during fetal life immediately before or during preterm birth, and in addition, later during postnatal life in a neonatal intensive care setting. During the prolonged hospital stay infection and inflammation might influence critical phases of myelination and cortical plasticity [4]. The association between perinatal infection and/or inflammation has been demonstrated by both epidemiological and experimental studies [5].

Nosocomial infections prolong hospital stay, increase hospital costs, and increase mortality [6, 7]. The vast majority of scientific literature dealing with nosocomial infections addresses bacterial or fungal infections, and viral agents are often disregarded. Civardi et al. [8] analyzed 590 neonatal infection outbreaks from the worldwide database of health care-associated outbreaks and 64 resulted from viruses, and 44 of which (68.75%) were reported from neonatal intensive care units.

Whether these nosocomial infections play a role for an increased risk for further infections remains to be investigated during the first 2 years of life. Less is known about the long-term consequences of nosocomial viral infections on either rehospitalization rates due to infections or neurocognitive development of VLBW infants during the first 2 years of life.

We aimed to determine the influence of nosocomial infection on neurodevelopmental outcome (NINO study) and on rehospitalization rates in VLBW preterm infants by means of a retrospective cohort study.

## Study design, methods and materials

**Fig. 1:**
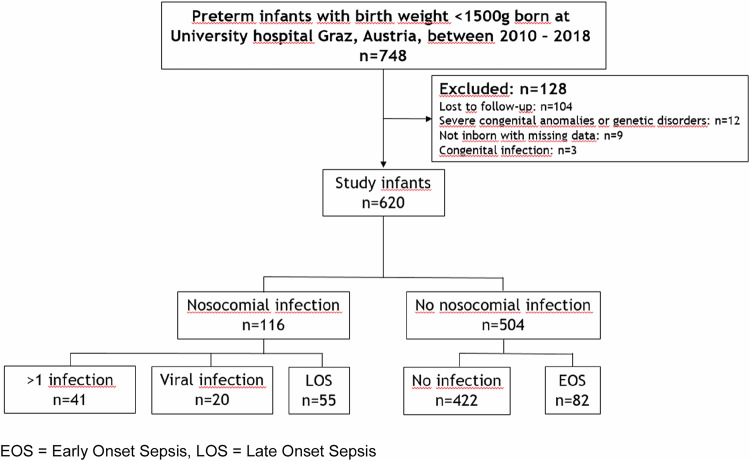
Flow-diagram depicturing presence of nosocomial infections in the study population (*n* = 620). Of 748 eligible infants 128 had to be excluded and of 620 study infants 116 had one or more nosocomial infection. More than 1 infection included 20 infants with EOS and LOS, 5 with EOS and viral infection, 12 with LOS and viral infection and 4 with EOS, LOS and viral infection.

The NINO study (Nosocomial Infection in VLBW infants ( < 1500 g): Influence on rehospitalization rate and Neurodevelopmental Outcome) is a retrospective observational cohort study including all preterm infants with birth weight below 1500  g born 2010–2018 and follow-up to the age corrected for prematurity of 2 years. Infants were stratified according to the presence or absence of nosocomial bacterial (late-onset sepsis) and viral infections. Cases were defined as preterm infants with late-onset sepsis or nosocomial viral infection; controls were those without nosocomial infections, but could have had early-onset sepsis; see Fig. 1.

Included were preterm very low birth weight (VLBW < 1500 g) infants with survival and follow-up until two years of age corrected for prematurity. Excluded were preterm infants with birth weight ≥1500 g, neonatal death, lost to follow-up, severe congenital anomalies or genetic disorders, connate infection, missing data and those outborn admitted at the age of more than 24 h.

Data were extracted from the local electronic patient management openMedocs® used at the University Hospital Graz. The local ethic committee of the Medical University of Graz ID 31-396 ex 18/19 approved the study. The study was registered at the “Deutsches Register Klinischer Studien” number DRKS00019000.

*Late onset sepsis* was defined as either clinical sepsis (at least 2 clinical signs and symptoms associated with elevated C-reactive protein (CRP) level above 10 mg/L or an immature-to-total neutrophil ratio >0.2 and negative blood culture, or blood culture proven sepsis - same as above and a positive blood culture with a plausible pathogen) and duration of antibiotic treatment for at least 7 days. Symptoms and signs of late-onset sepsis had to be observed after the third day of life ( > 72 h postpartum):

- temperature instability: hyperthermia >38.5 °C, hypothermia <36.0 °C, or undulations of 1.5 degree Celsius over 24 h
- respiratory disorders: tachypnea >60/min, dyspnea (nasal flares, retractions, grunting), apnea
- gastrointestinal symptoms: drinking weakness, vomiting, abdominal distension
- circulatory insufficiency: tachycardia >180 bpm, bradycardia <80 bpm, arterial hypotension needing volume or inotropes, prolonged capillary filling time >2 sec, paleness
- metabolic acidosis, hyperglycemia or hypoglycemia
- oliguria or anuria
- neurological disorders: hyperexcitability, convulsive seizures
- septic shock, multi organ failure, disseminated intravascular coagulation

*Nosocomial viral infections* were defined as respiratory or gastrointestinal infections acquired during the first hospital stay. Nasopharyngeal swaps and stool cultures were done routinely in case of a symptomatic preterm infant. No invasive fungal infections were documented during the study period (a prophylactical antifungal therapy was given in all VLBW infants).

*Maternal data* included maternal age, mode of delivery and morbidities including preeclampsia, pregnancy-induced hypertension (PIH), HELLP syndrome (hemolysis + elevated liver enzymes + low platelet count), preterm premature rupture of membranes (PPROM) and amniotic infection syndrome (AIS).

*Perinatal data* included gestational age in weeks, birth weight in grams including small for gestational age (SGA)/ intrauterine growth restriction (IUGR), gender, APGAR score at 1, 5, and 10 min after birth, and multiple pregnancy (twins or triplets).

*Neonatal data* included respiratory distress syndrome (RDS with surfactant application), bronchopulmonary dysplasia (BPD), Ureaplasma or Mycoplasma pneumonia, patent ductus arteriosus (PDA), intra-periventricular hemorrhage (I/PVH), periventricular leukomalacia (PVL), ileus, intestinal perforation, necrotizing enterocolitis (NEC), retinopathy of prematurity (ROP), neonatal abstinence syndrome (NAS), and the ventilation mode (invasive/ INSURE [intubate-surfactant-extubate] or non-invasive), duration of ventilation and duration of neonatal hospital stay.

***Follow-up data*** included all admissions due to infectious diseases during the first 2 years of life, the number of rehospitalizations per infant, the age in months (chronological age) at time of rehospitalization and the duration of each hospital stay. The infections were subdivided into respiratory tract infection, gastrointestinal tract infection, and other infectious diseases (febrile infection without focus, febrile seizures, urinary tract infection, wound infection, roseola infantum, pharyngitis, tonsillitis, otitis media, nephrolithiasis, lymphadenitis, aphthous stomatitis, preseptal cellulitis, encephalitis, meningitis).

**N*eurodevelopmental outcome*** at two years of age corrected for prematurity was done using the German adaptation of Bayley Scales of Infant Development (BSID) II (second edition published in 2007) and the German adaption of the BSID III (published in 2014). The BSID II was used until 2015 and the BSID III thereafter. We assessed the mental development index (MDI) and physical development index (PDI) when using the BSID II, and the cognitive and motor scales when using the BSID III (the language scale was not part of the test battery at our institution, speech and language assessment using SETK-2 was conducted at the age of 2.5 years).

The reliability coefficients of subtests of the Bayley-III-scales are between *r* = 0.77 and *r* = 0.89; the reliability coefficients of the scales between *r* = 0.86 and *r* = 0.88; and the mean reliability coefficients of subtests between *r* = 0.68 and *r* = 0.83. The standardization of Bayley-III included 878 German children without any known impairment, split in 17 age groups, adding 131 Dutch infants; a total sample of 1009 children.

Some infants could not be tested due to their cognitive or motor impairment. Therefore, we introduced an additional definition of the neurodevelopmental impairment (NDI), which depended on the detailed descriptions in the medical documents at the age of 2 years corrected for prematurity [9]. The term NDI represents neurologic disability defined by one or more of the following impairments: infants with cerebral palsy with a gross motor function classification system (GMFCS) level ≥2, a BSID II or III cognitive score of <70, severe visual impairment (for example blindness, optic atrophy, central vision disorder) or severe hearing impairment with need of hearing aids. We did a secondary analysis of moderate and severe NDI by using different cognitive scores. Moderate NDI was defined as a score <85 and severe NDI as a cognitive score <70.

***Statistical analyses*** were done using IBM SPSS Statistics 26. Data are presented as mean ± standard deviation (SD) or median and interquartile range for continuous variables and absolute and relative frequencies for categorical data. To identify predictors for neurodevelopmental impairment and rehospitalization uni- and multivariate logistic regression analyses were performed. Variables with a *p* < 0.1 were considered for multivariate analysis. Before calculating multivariate logistic regression analysis, potential predictors were checked for multicollinearity. Odds ratios with 95% CI and *p*- values are reported for uni- and multivariate results.

Exploratory, the impact of nosocomial infections on cognitive and motor outcome, using different thresholds ( < 70 vs. ≥70; <85 vs. ≥85), cognitive NDI ( < 85 vs. ≥85), NDI vis/hear/phys (yes/no), visual disability (yes/no), hearing disability (yes/no) and physical disability (yes/no) was analysed using chi square test or Fisher’s exact test, as appropriate, and the difference in cognitive outcome and motor outcome was analysed using *t* test. Statistics

## Results

### Study group data

During the study period 2010–2018, we treated 748 preterm infants with birth weight below 1500 g. One hundred and twenty-eight infants (17%) had to be excluded: 104 were lost to follow-up, 12 had severe congenital malformation, 9 were not born at the University Hospital of Graz and/or had missing data, and 3 had congenital infections. Thus, the study group comprised 620 infants.

The maternal and perinatal data of the study population are given in Table 1, the neonatal data in Table 2. Table 3 shows the neurodevelopmental outcome data of the study population. The group of 104 preterm infants without follow-up did not differ regarding neonatal complications (IVH/PVH, cystic PVL, NEC, retinopathy and bronchopulmonary dysplasia).

**Table 1:** Maternal and perinatal data of 620 preterm infants <1500 g born at the University Hospital Graz, Austria, between 2010 and 2018.

Parameters	Results
Maternal age	31 ± 5.7 (15–50)
PPROM	185 (30)
Chorioamnionitis	60 (9.7)
Preeclampsia	123 (20)
Cesarean section	552 (89)
Gestational age in weeks	29 ± 2.7 (23–37)
Gestational age >32 weeks	45 (7.3)
Birth weight in grams	1095 ± 285 (355–1499)
Birth weight below 1000 g	221 (36)
Small For Date ( < 10.percentile)	167 (27)
APGAR Score at 1 min	7 ± 1.9
APGAR Score at 5 min	8 ± 1.2
APGAR Score at 10 min	9 ± 0.9
Multiples	216 (35)
Twins / Triplets	191 (88) / 25 (12)
Male gender	295 (47)

Data are given as n (%) or mean ± SD (range).

**Table 2:** Neonatal data of 620 preterm infants <1500 g born at the University hospital Graz, Austria, between 2010 and 2018.

Parameters	Results
Respiratory distress syndrome	522 (84)
Mechanical Ventilation	555 (90)
Non-Invasive Ventilation	188 (34)
Duration of Ventilation (days)	30 (1-327)
Duration of hospitalization (days)	64 (11-327)
Patent ductus arteriosus, hemodynamically significant	198 (32)
Intraventricular Hemorrhage Grade 1–2	74 (12)
Intraventricular Hemorrhage Grade 3	16 (3)
Periventricular Hemorrhage	11 (2)
Cystic periventricular Leukomalacia	15 (2)
Ileus	32 (5)
Intestinal Perforation	15 (2)
Necrotizing Enterocolitis	14 (2)
Retinopathy of Prematurity Grade 3–5	22 (4)
Bronchopulmonary Dysplasia	1 (0.2)

Data are given as n (%) or median (range).

**Table 3:** *Neurodevelopmental outcome* data of 620 preterm infants <1500 g born at the University Hospital Graz, Austria, between 2010 and 2018.

Parameters	Results
Neurodevelopmental impairment	202 (32.6)
Cognitive score <70	33 (5.3)
Cognitive score <85	89 (14.4)
Motoric score <70	14 (2.3)
Motoric score <85	72 (11.6)
Visual impairment	6 (0.97)
Hearing impairment	4 (0.65)
Physical impairment	23 (3.7)
Total cognitive outcome	100 ± 17.8 (55–145)
Total motor outcome	95 ± 12.4 (49–128)

Data are given as n (%) or mean ± SD (range).

### Neurodevelopmental impairment

Of 620 study patients 418 had no NDI and were compared to 202 infants with NDI according to the predefined criteria. Rates of nosocomial infections differed significantly between groups (4.8 versus 10.4%, see Table 4). Groups did not differ regarding rates of early-onset sepsis. Infants with NDI were of younger gestational age, had lower birth weights and higher rates of neonatal complications (cystic PVL, I/PVH). They had longer neonatal stays at the hospital and more long-term morbidities (ROP and BPD). Multivariate analysis revealed neonatal ileus, PVL and I/PVH significantly associated with NDI after checking for multicollinearity. Comparing rates of NDI in infants with nosocomial infections excluding cases with early-onset sepsis to infants without infections revealed no differences (37.9% [33/87] vs. 30.3% [128/422], *p* = 0.072). The distribution of infectious diseases is shown in Fig. 1 presenting 116 infants with one or more nosocomial infections and 504 without (the latter included 82 [16.3%] infants with early-onset sepsis). The comparison of NDI in infants with early-onset sepsis to those without did not reveal differences as did not a single versus multiple nosocomial infections. Groups with infectious complications were partly very small (early-onset sepsis + viral infection + late-onset sepsis, *n* = 4; late-onset sepsis + viral, *n* = 12; early-onset sepsis + viral, *n* = 5). Infants with two or more infections including early-onset sepsis had lower cognitive and motor scores, and the rate of moderate motoric impairment ( < 85) also differed significantly (Table 3). Comparison of NDI in infants without and those with any kind of infection revealed significant differences - 128/422 infants (30.3%) compared to 76/198 (38.4%), *p* = 0.02. The latter were of younger gestational age and had lower birth weights median 30–27 weeks and 1235–934 g, *p* < 0.001, respectively.

**Table 4:** Comparison 418 preterm infants <1500 g without and 202 with neurodevelopmental impairment born at the University Hospital Graz, Austria, between 2010 and 2018.

	*NDI*		*univariate*		*multivariate*	
*Parameter*	*No (n* = *418)*	*Yes (n* = *202)*	*OR (95%CI)*	*p value*	*OR (95%CI)*	*p value*
Gender						
male	190 (46)	103 (51)	1.25 (0.89–1.75)	0.196		
female	228 (55)	99 (49)	ref.			
Gestational age in weeks	29 (27–31)	28 (26–30)	0.91 (0.85–0.97)	0.002		
Birth weight in grams	1180 (938–1368)	1038 (760–1290)	0.999 (0.997–0.999)	<0.001		
Noscomial infections ( > 1)	20 (4.8)	21 (10.4)	2.31 (1.22–4.37)	0.010		
Early-onset sepsis (EOS)	69 (17)	42 (21)	1.33 (0.87–2.04)	0.193		
IRDS	340 (81)	182 (90)	2.09 (1.24–3.52)	0.006		
Bronchopulmonary Dysplasia	22 (5)	31 (15)	3.26 (1.84–5.80)	<0.001	2.94 (1.62–5.32)	<0.001
Patent Ductus Arteriosus	125 (30)	73 (36)	1.33 (0.93–1.89)	0.119		
Intraventricular Hemorrhage ( = IVH)				0.002		
no IVH	369 (88)	161 (80)	ref.			
Grade 1–3	49 (12)	41 (20)	2.82 (1.36–5.84)	0.005		
Periventricular Hemorrhage	4 (1)	7 (4)	9.27 (2.58–33.27)	0.001		
Periventricular Leukomalacia ( = PVL)				<0.001		<0.001
no PVL	401 (96)	173 (86)	ref.			
Grade 1	14 (3)	17 (8)	2.82 (1.36–5.84)	0.005	2.43 (1.14–5.18)	0.021
Grade 2–4	3 (1)	12 (6)	9.27 (2.58–33.27)	0.001	8.85 (2.41 32.5)	0.001
Ileus	12 (3)	20 (10)	3.72 (1.78–7.77)	<0.001	2.92 (1.35–6.33)	0.007
Intestinal Perforation	6 (1)	9 (5)	3.20 (1.12–9.12)	0.029		
Necrotizing Enterocolitis	6 (1)	8 (4)	2.83 (0.97–8.27)	0.057		
Retinopathy of Prematurity ( = ROP)				0.023		
no ROP	333 (80)	147 (73)	ref.			
Grade 1–2	76 (18)	41 (20)	1.22 (0.80–1.87)	0.357		
Grade 3–5	9 (2)	13 (7)	3.27 (1.37–7.82)	0.008		
Ventilation	366 (88)	189 (94)	2.07 (1.10–3.89)	0.025		
Non-Invasive Ventilation	132 (32)	56 (28)	0.83 (0.57–1.20)	0.328		
Invasive Ventilation/ INSURE	234 (56)	133 (66)	1.52 (1.07–2.15)	0.001		
Days of Ventilation	13 (4-39)	28 (5-56)	1.01 (1.01–1.02)	<0.001		
Days of neonatal stay at the hospital	53 (37-72)	65 (46-98)	1.02 (1.01–1.02)	<0.001		

Data are given as n (%) or mean ± SD (range).

### Pathogens

Bacterial pathogens identified in nosocomial infections included Staphylococcus epidermidis, Staphylococcus hemolyticus, Enterococcus faecalis, Enterococcus faecium, Staphylococcus aureus, Staphylococcus warneri, Staphylococcus hominis, and Klebsiella pneumoniae. Viruses included Rotavirus, Rhinovirus, respiratory syncytial virus (RSV), Adenovirus, Norovirus, Influenza A virus, Boccavirus, and Coronavirus in descending order of frequency. Due to heterogeneity of pathogens no further analyses were calculated.

### Rehospitalizations within first two years

A total of 205 infants (33%) were rehospitalized during their first two years of age. Twenty-seven of them (13.2%) had had nosocomial infections. Those with vs. those without nosocomial infection experienced 277 vs. 117 episodes of infectious diseases, resulting in 2.6 vs. 1.7 rehospitalizations per infant (*p* = 0.002). Age distribution between groups showed those with a history of nosocomial infections had rehospitalizations at older age ( > 12 months 38% vs. 56%, *p* < 0.001). Between the age of 6 and 12 months there were no differences (35 vs. 34%), but more hospitalizations at the age below six months (27 vs. 10%, *p* < 0.001). Despite these differences, multivariate analyses revealed only low gestational age as a significant predictor for rehospitalizations during the first two years of life (see Table 5).

**Table 5:** Rehospitalizations in 205 out of 620 preterm infants <1500 g born at the University Hospital Graz, Austria, between 2010 and 2018 and risk factors analysis.

	*Rehospitalisation*		*univariate*		*multivariate*	
*Parameter*	*No (n* = *415)*	*Yes (n* = *205)*	*OR (95%CI)*	*p value*	*OR (95%CI)*	*p value*
Gender						
male	192 (46)	101 (49)	1.13 (0.81–1.58)	0.481		
female	223 (54)	104 (51)	ref.			
Gestational age in weeks	29 (27–31)	28 (26–30)	0.90 (0.84–0.96)	0.001	0.90 (0.84–0.96)	0.001
Birth weight in grams	1160 (900–1368)	1060 (814–1300)	0.999 (0.998–1.000)	0.002		
Noscomial infections ( > 1)	27 (7)	14 (7)	1.05 (0.54–2.06)	0.879		
Early-onset sepsis (EOS)	74 (18)	37 (18)	1.02 (0.66–1.57)	0.947		
IRDS	344 (83)	178 (87)	1.36 (0.84–2.20)	0.207		
Bronchopulmonary Dysplasia	25 (6)	28 (14)	2.47 (1.40–4.35)	0.002		
Patent Ductus Arteriosus	127 (31)	71 (35)	1.20 (0.84–1.71)	0.311		
Intraventricular Hemorrhage ( = IVH)						
no IVH	360 (87)	170 (83)	ref.			
Grade 1–3	55 (13)	35 (17)	1.35 (0.85–2.14)	0.205		
Periventricular Hemorrhage	6 (1)	5 (2)	1.70 (0.51–5.65)	0.383		
Periventricular Leukomalacia ( = PVL)				0.151		
no PVL	382 (92)	192 (94)	ref.			
Grade 1	25 (6)	6 (3)	0.48 (0.19–1.18)	0.110		
Grade 2–4	8 (2)	7 (3)	1.74 (0.62–4.87)	0.291		
Ileus	18 (4)	14 (7)	1.62 (0.79–3.32)	0.191		
Intestinal Perforation	11 (3)	4 (2)	0.73 (0.23–2.32)	0.595		
Necrotizing Enterocolitis	8 (2)	6 (3)	1.53 (0.53–4.48)	0.434		
Retinopathy of Prematurity ( = ROP)				0.006		
no ROP	336 (81)	144 (70)	ref.			
Grade 1–2	68 (16)	49 (24)	1.68 (1.11–2.55)	0.014		
Grade 3–5	10 (2)	12 (6)	2.80 (1.18–6.63)	0.019		
Ventilation	364 (88)	191 (93)	1.91 (1.03–3.54)	0.040		
Non-Invasive Ventilation	129 (31)	59 (29)	0.90 (0.62–1.29)	0.557		
Invasive Ventilation/ INSURE	235 (57)	132 (64)	1.39 (0.98–1.96)	0.065		
Days of Ventilation	13 (4 – 42)	25 (4 – 48)	1.01 (1.00–1.01)	0.003		
Days of neonatal stay at the hospital	54 (36 – 74)	91 (44 – 90)	1.01 (1.00–1.01)	<0.001		

Data are given as n (%) or mean ± SD (range).

## Discussion

Results demonstrated that nosocomial infections did not significantly influence NDI. Early-onset sepsis alone also did not significantly increase the rate of NDI. Hence, comparison of groups without any infection and those with two or more resulted in lower cognitive and motor scores at the corrected for prematurity age of two years. Our hypothesis was not true that one or more nosocomial infectious hits might increase the rate of neurocognitive sequelae. In contrast, classical complications of neonatal intensive care including BPD, cystic PVL and ileus were stronger risk factors for NDI than nosocomial infections.

Five years’ outcome data from the Epipage study reported on a 1.7-fold increased risk developing cerebral palsy with either isolated early- or late-onset sepsis. And a 2.3-fold increased risk has been reported in case of diagnosis of both early- and late-onset sepsis [10]. Earlier studies by Stoll et al. [11] revealed that infections increased the risk of neurodevelopmental impairment and the rate of cerebral palsy by factor 1.4–1.7. The type of infectious pathogen had little or no influence on the outcome of the infants. Stoll et al. [11] additionally demonstrated that infections negatively influenced weight gain and head circumference. Over a nearly 7-year time period Zonnenberg et al. [12] analyzed 85 of 117 VLBW infants with suspected late-onset sepsis who experienced blood culture–proven infection. Coagulase-negative staphylococci were responsible for 55% of the episodes. They found no differences regarding outcome of the infants by comparing infants with versus no proven infection. A comparison of three groups – those with coagulase-negative staphylococci, those with other bacteria, and infants with negative blood culture—the composite cognitive scores differed significantly in favor for the coagulase-negative staphylococci group versus other causal agent group. There were no differences concerning other subscales [12]. A multicenter retrospective cohort study including 3940 infants with a gestational age of 22–26 weeks between 2006 and 2014 revealed a higher adjusted relative risk for death in case of culture proven late-onset sepsis [13]. By protocol these infants did not have early-onset sepsis, NEC or an episode of culture negative late-onset sepsis. The risk for NDI among survivors at the age of 18–26 months did not differ between infants with and those without culture proven late- onset sepsis, but the latter had a higher risk for NDI compared to unaffected infants. A study on 203 infants with gestational age 24–32 weeks assessed brain injury from MRI records and evaluated neurodevelopmental outcome at 36 months [14]. Infants were stratified in those without sepsis, cases with EOS and cases with LOS. The authors found a higher prevalence of cerebellar hemorrhage in infants with late-onset sepsis and lower motor, cognitive, and language composite scores. After adjusting for gestational age, birth weight, cerebellar hemorrhage, and white matter injury, only the motor scores remained significant. Our study showed that low gestational age leading to neonatal complications including I/PVH, PVL and neonatal ileus were stronger risk factors for NDI by multivariate analysis than nosocomial infections. Nosocomial infection rates were significantly higher in the group with NDI by univariate analysis. Hence, it remains difficult to outweigh the individual risk factors profile being associated with NDI or to what extent nosocomial infections compromise extremely low gestational age infants.

In a small pilot study using near infrared spectroscopy in a cohort of very low birth weight infants with late-onset sepsis (predominantly coagulase negative staphylococci) the authors detected no cerebral hypoxemia as a consequence of inflammation [15]. By follow-up at the age of two years no differences were found between groups. Thus, NIRS might not be the right tool to search for reasons for the worse outcome of preterm infants having had nosocomial infections.

Hence, inflammatory processes maybe last longer over a certain period of postnatal life in the surrounding of the neonatal intensive care unit and negatively influence critical phases of myelination and cortical plasticity [3]. Neuroinflammation during the perinatal period thus increases the risk of neurological and neuropsychiatric disease from childhood to adulthood. Longitudinal measures of inflammation and neurobehavior in 73 very preterm infants showed associations between IL-1 receptor antagonist and motor development, IL-6 and alertness and orientation scores, and tumor necrosis factor-alpha (and composite scores) with motor development and vigor and alertness/ orientation scores [16].

Experimental studies have shown a causal effect of infection/inflammation on perinatal brain damage [4]. Infection including inflammatory factors can disturb processes of brain development and induce death and/or blockade of oligodendrocyte maturation, leading to myelin defects. This is especially the case between 28 and 32 weeks of gestational age [17]. The so-called multiple-hit hypothesis alternatively recognizes infection and/or inflammation as predisposing factors that make the brain more susceptible to a second stress [18]. Data also suggest that perinatal exposure to inflammatory factors may have long-term sequelae regarding diseases in adulthood including psychiatric disorders [4].

Preterm infants are prone to infection throughout their neonatal hospitalization. The developmental processes of the brain are enormous during the stay at the NICU, and thus, cytokines and other proinflammatory proteins and enzymes have many possibilities to damage the vulnerable white matter of the brain. In this scenario, developmentally regulated processes involving the central nervous and respiratory systems might become disturbed or disrupted. As a result, infants are at increased risk for death [19], chronic lung disease, and adverse neurodevelopmental outcome [18].

The role of nosocomial viral infections is neither well characterized and nor its effects on the developing brain. Civardi et al. [8] described sixty-four outbreaks caused by viruses, with the five most common viral agents being rotavirus (23%), respiratory syncytial virus (17%), enterovirus (16%), hepatitis A virus (11%), and adenovirus (9.4%). The fact that nosocomial infections might pave the way to an increased risk for later viral infections was not proven by our study. The immunological reasons and mechanisms might simply rely to the extremely low gestational age.

Perinatally acquired viral infections have been increasingly reported to be associated with an adverse neurological outcome [20]. The white matter is predominantly involved, but the exact pathophysiology is not known and therapeutic options are limited. A meta-analysis of 18 studies including 13.755 very preterm (VLBW) infants with perinatal infections had poorer mental and motor development compared to those without infections [21]. Postnatal infections and especially NEC and not chorioamnionitis were found to be responsible for detrimental effects on neurodevelopmental outcome.

Strength of our study include the large number of carefully analysed VLBW infants with follow-up investigation in a region of approximately 9.000 births per year covering all rehospitalizations and follow-up examinations. There are certain limitations that have to be mentioned including the retrospective study design and the small numbers of preterm infants in the group with two or more nosocomial infections.

In conclusion we found that nosocomial infections did not significantly influence NDI and rehospitalization rates due to infectious diseases. Risk factors associated with higher rates of NDI included BPD, cystic PVL and ileus. Low gestational age was associated with higher rates of rehospitalizations.

## Data Availability

The datasets generated during and/or analysed during the current study are available from the corresponding author on reasonable request.
